# Community initiated kangaroo mother care and early child development in low birth weight infants in India-a randomized controlled trial

**DOI:** 10.1186/s12887-020-02046-4

**Published:** 2020-04-04

**Authors:** Sunita Taneja, Bireshwar Sinha, Ravi Prakash Upadhyay, Sarmila Mazumder, Halvor Sommerfelt, Jose Martines, Suresh Kumar Dalpath, Rakesh Gupta, Patricia Kariger, Rajiv Bahl, Nita Bhandari, Tarun Dua, for the ciKMC development study group, Farah Abbasi, Vaishali Panwar, Sugandhi Nagpal, Medha Shekhar, Runa Ghosh, Jasmine Kaur, Brinda Dube

**Affiliations:** 1 Centre for Health Research and Development, Society for Applied Studies, 45, Kalu Sarai, New Delhi, 110016 India; 2 Department of Paediatrics, Faculty of Medicine and Health Technology, Tampere University, Tampere, Finland; 3 Department of Global Public Health and Primary Care, University of Bergen, Bergen, Norway; 4 Centre for Intervention Science in Maternal and Child Health, Department of Global Public Health and Primary Care, University of Bergen, Bergen, Norway; 5 Norwegian Institute of Public Health, Oslo, Norway; 6 State Health System Resource Centre, Haryana, India; 7 Department of School Education, Government of Haryana, Panchkula, India; 8 Center for Effective Global Health, University of California, Berkeley, USA; 9 Department of Maternal, Newborn, Child and Adolescent Health, World Health Organization, Geneva, Switzerland; 10 Department of Mental Health and Substance Abuse, World Health Organization, Geneva, Switzerland

**Keywords:** Child development, Kangaroo mother care, Low birth weight, community initiated

## Abstract

**Background:**

In a randomized controlled trial (RCT) with 8402 stable low birthweight (LBW) infants, majority being late preterm or term small for gestational age, community-initiated KMC (ciKMC) showed a significant improvement in survival. However, the effect of ciKMC on neurodevelopment is unclear. This is important to elucidate as children born with low birth weight are at high risk of neurodevelopmental deficits. In the first 552 stable LBW infants enrolled in the above trial, we evaluated the effect of ciKMC on neurodevelopmental outcomes during infancy.

**Method:**

This RCT was conducted among 552 stable LBW infants, majorly late preterm or term small for gestational age infants without any problems at birth and weighing 1500–2250 g at birth. The intervention comprised of promotion of skin-to-skin contact and exclusive breastfeeding by trained intervention delivery team through home visits. The intervention group mother-infant-dyads were supported to practice ciKMC till day 28 after birth or until the baby wriggled-out. All infants in the intervention and control groups received Home Based Post Natal Care (HBPNC) visits by government health workers. Cognitive, language, motor and socio-emotional outcomes were assessed at infant-ages 6- and 12-months using Bayley Scale of Infant Development (BSID-III). Other outcomes measured were infant temperament, maternal depression, maternal sense of competence, mother-infant bonding and home-environment. We performed post-hoc equivalence testing using two one-sided tests of equivalence (TOST) to provide evidence that ciKMC does not do harm in terms of neurodevelopment.

**Results:**

In the intervention arm, the median (IQR) time to initiate ciKMC was 48 (48 to 72) hours after birth. The mean (SD) duration of skin-to-skin-contact was 27.9 (3.9) days with a mean (SD) of 8.7 (3.5) hours per day. We did not find significant effect of ciKMC on any of the child developmental outcomes during infancy. The TOST analysis demonstrated that composite scores for cognitive, language and motor domains at 12 months among the study arms were statistically equivalent.

**Conclusion:**

Our study was unable to capture any effect of ciKMC on neurodevelopment during infancy in this sample of stable late preterm or term small for gestational age infants. Long term follow-up may provide meaningful insights.

**Trial registration:**

The trial is registered at clinicaltrials.gov NCT02631343 dated February 17, 2016; Retrospectively registered.

## What this paper adds

- Probably the first trial to document the effect of KMC initiated at home (community initiated KMC; ciKMC) on neurodevelopmental outcomes in a selected sample of stable low birth weight infants.
- No significant discernible benefit of ciKMC on neurodevelopment at 12 months of corrected age
- Long-term follow-up may provide meaningful insights

## Background

Low birthweight (LBW) infants have higher rates of mortality, morbidity, growth and cognitive impairment compared to infants with birth weight ≥ 2500 g [1–4]. Current strategies recommend prioritization of interventions that impact both survival and development (“Survive and Thrive”). Kangaroo Mother Care (KMC) is a novel intervention known to improve survival, nutrition and prevent infections [5].

KMC encompasses prolonged skin-to-skin contact (SSC) between the mother and the baby and exclusive breastfeeding (EBF) till the end of neonatal period or until the baby wriggles out, whichever is earlier [6]. A Cochrane systematic review indicates that compared to conventional care, KMC in LBW babies in hospitals reduces deaths (RR 0.67, 95% CI 0.48, 0.95) and risk of severe infection or sepsis (RR 0.50, 95% CI 0.36, 0.69). A 72% reduced risk of hypothermia at discharge or 40–41 weeks postmenstrual age, and benefits on weight and length gain were also observed [5].

Pathways through which KMC could improve neurodevelopmental outcomes includes optimal nutrition through breastfeeding, reducing severe infections, promoting stimulation and improving maternal responsiveness and mother-infant interaction [7]. Some available evidence is suggestive of short as well as long-term benefits of hospital-initiated KMC on child development [8–14]. In preterm infants receiving KMC in the health facility, studies indicate better child developmental outcomes [11, 15, 16]. In an intervention study where preterm infants were followed up 20 years after enrollment (~ 60% could be tracked), the young adults who received KMC in infancy had reduced school absenteeism, less hyperactivity, aggressiveness, externalization, and socio-deviant conduct, compared to the controls who did not receive KMC [12].

In India, even though the proportion of institutional births is increasing, a sizeable number of births still occur at home. These newborns have limited access to quality health care services or hospital-based interventions which can potentially improve survival, growth and development. Moreover, a high proportion of babies born in facilities, even those with LBW, are discharged within a day or two of being born and therefore the caregivers have limited opportunity to learn the technique and benefits of KMC. In 2014, the Government of India endorsed initiation of KMC in health facilities for LBW infants [17]. However, in most facilities in India, KMC is either poorly or not implemented [18, 19]. Therefore, initiating KMC at the home/community level (community initiated KMC; ciKMC), through trained community health workers, seems promising as a strategy to improve coverage of this live-saving intervention.

Globally, there is limited evidence on the benefits of ciKMC. A study in Bangladesh, designed to test the mortality impact of ciKMC, was not conclusive, but provided useful insights into the barriers of implementing KMC within the community [20]. Recently, a large community-based randomized controlled trial in North India showed a substantial improvement in neonatal survival and infant survival up to 6 months age in stable LBW infants, majorly late preterm or term small for gestational age infants, without any problems at birth and weighing 1500–2250 g at birth, as an effect of ciKMC [21]. However, the effects of ciKMC on neurodevelopment outcomes in LBW infants are still unclear. Therefore, in the first 552 of the infants enrolled in the above trial, our objective was to assess the impact of ciKMC on neurodevelopmental outcomes at 6 and 12 months of age and on maternal depressive symptoms; maternal sense of competence; mother-infant attachment and home environment.

## Method

### Study design and participants

This was an individually randomized unmasked controlled trial (RCT) conducted between July 2015 to November 2016. The first 552 infants enrolled in the primary trial [21] were included for evaluation of child developmental outcomes. This cohort of LBW infants was a very selected cohort consisting majorly of stable late preterm or term small for gestational age infants without any problems at birth. Although the inclusion weight was 1500–2250 g, yet for those weighing between 1500 and 1800 g referral was facilitated for hospital care following Government of India guidelines. The infants weighing between 1500 and 1800 g were considered for inclusion only if the families refused to take the baby to the hospital, or if the baby was taken to hospital but was either not admitted, or admitted and discharged before s/he became 72 h old and not started on KMC. Infants unable to feed, with difficulty in breathing, with less than normal movements or gross congenital malformations, those for whom KMC was initiated in hospitals, and those whose mothers did not intend to stay in the study area for the next 6 months or did not consent to participate were excluded. The weight category was determined based on our formative research findings [22] that suggested most babies with birth weight > 2250 g wriggle out of KMC position before the neonatal period. The lower cut-off 1500 g was considered to avoid including infants who would have been at a high-risk of complications and would have required hospital care. The study was conducted in rural and semi-urban populations of around 2 million in Haryana, North India. In the study sites, around 40% were home births and around one-fourth of all babies were born with LBW. Details of the study settings have been published elsewhere [23].

### Enrollment, randomization and allocation

Ethical clearances were obtained from the Institutional Ethics Review Committee and the WHO Ethics Review Committee. State approvals were also available. Pregnant women were identified by a door-to-door pregnancy surveillance team every three months. Identified pregnant women were followed-up regularly till delivery, with the frequency of contacts being higher in the third trimester. Newborns were visited at home and weighed as early as possible. A digital hanging weighing scale (AWS-SR-20; American Weigh Scales, Cumming, GA, USA) was used for weight measurement. Gestational age was documented from ultrasound report, hospital records or maternal recall, whichever was available, in the given order of preference. After screening as per inclusion and exclusion criteria, in the eligible mother-infant dyads, a study worker obtained written informed consent in the local language from caregivers prior to enrollment.

The unit of randomization was the mother-infant dyad. The randomization list was prepared by an independent statistician using random permuted blocks of variable size. Allocation of study identification number was done by an off-site randomization coordinator using serially numbered opaque sealed envelopes (SNOSE), kept under lock and key. Similar procedures were followed for participants enrolled in intervention or in the control group. If a dyad was allocated to the intervention (ciKMC) group, the randomization coordinator subsequently informed the intervention delivery team. We attempted to ensure that the study team responsible for outcome assessment and study investigators were not aware of the group allocation till the end of the study.

### Intervention delivery

The ciKMC intervention comprised of promotion and support of skin-to-skin contact and exclusive breastfeeding by the intervention workers and supervisors. Mother-infant dyads allocated to the ciKMC group were visited at home by a trained intervention delivery team consisting of a pair of workers as soon as possible after enrollment to explain and initiate KMC and support its practice. The team home-visited daily for the first 3 days, then on days 5 and 7, twice in the second week and once each in the 3rd and 4th week to provide support and solve any problems related to practicing KMC. During home visits, the team observed the mother practicing KMC, enquired about skin-to-skin contact and breastfeeding in the preceding 24-h period, and supported the mother and family to solve any problems or overcome barriers to effective KMC. They counselled that skin-to-skin-contact be done for as long as possible during day and night, preferably for 24 h a day, with the assistance of other family members. Visits continued till 28 days of age or until the baby wriggled out and no longer accepted SSC, whichever was earlier. The intervention delivery was designed based on previous formative research [22]. All infants in the intervention and control groups received Home Based Post Natal Care (HBPNC) visits by government health workers (Accredited Social Health Activists; ASHAs) as implemented through the health system [24]. Specific details of the intervention have been published elsewhere [23].

### Outcomes

The outcomes were cognitive, language, motor, socio-emotional development and infant temperament scores at 6 and 12 months of infant age.; maternal depressive symptoms at 6 weeks and 6 months of infant age; maternal sense of competence at 6 weeks and 12 months of infant-age; mother-infant attachment at 6 weeks of infant-age and assessment of the home environment at 12 months of infant-age.

### Outcome ascertainment

Information on skin-to-skin-contact (number of days and average hours per day), as reported by the mother, in both the groups was ascertained by a trained outcome ascertainment team at the end of neonatal period. Developmental outcomes were ascertained in the study clinic by trained psychologists, who were unaware of allocation.

Bayley Scales of Infant and Toddler Development, 3rd Edition (BSID-III) was used to ascertain child development (cognition, language, motor and socio-emotional performance) at 6 and 12 months of age corrected for gestational age [25]. We adapted BSID-III for use in the study setting. For the adaptation, the test items were reviewed by the team of psychologists and public health experts in terms of cultural relevance, and subsequently, modifications were identified, discussed and incorporated. While conducting the adaptations, care was taken to match the style of the original item. For items that required translation in the local language i.e. Hindi, the translation was done by psychologists fluent in the local language and with a thorough understanding of the cultural context. An individual fluent in English language, and not a part of the study team, performed the back-translation. Prior to the start of the formal testing, the adapted materials were piloted on approximately 15–20 infants who were not a part of the trial.

The infant temperament scale was used as adapted in the MAL-ED study [26]. This 47 item-scale covered six domains i.e. activity, positive emotionality, negative emotionality, sociability, attention and soothability, where higher scores reflect more difficult temperament. Maternal depressive symptoms were assessed using the Patient Health Questionnaire (PHQ)-9; higher scores reflecting more depressive symptoms. The PHQ-9 is the depression module of the self-administered version of the PRIME-MD diagnostic instrument. The 9 items of PHQ-9 tool are based on the DSM-IV diagnostic criteria [27].

Maternal sense of competence was assessed using “maternal self-efficacy scale” that consists of 10 questions with four point scale responses; higher scores reflecting better maternal self-efficacy [28]. The maternal postnatal attachment scale was used to assess mother-infant attachment. This scale consists of 19 items with higher scores reflecting better attachment [29]. Home environment was assessed using “Pediatric Review of Children’s Environmental Support and Stimulation (PROCESS)” questionnaire. It consisted of three components: clinical observation, parent questionnaire and toy list. Higher scores reflect better stimulation and support to infants [30]. The study questionnaires were adapted according to local cultural context, translated in local language (Hindi), pre-tested and validated for use.

### Sample size

To examine a difference of 0.25 SD between the intervention and the control group for cognitive, language, motor and socio-emotional outcomes at 80% power, 260 infants in each group i.e. 520 infants were required. 552 infants were enrolled, assuming a 10% loss to follow up.

### Statistical analysis

All analysis was done using STATA version 14.0 (Stata Corp, Texas, USA). Intention to treat analysis was performed. The distribution of continuous data was examined using histograms and skewness and kurtosis coefficients calculated. Mean (SD; standard deviation) or median (IQR; inter-quartile range) were calculated for continuous variables and proportions for categorical variables. Distribution of baseline data on household, maternal and paternal, birth-related and infant characteristics were compared across the intervention and control groups. Chi-square test was used to compare proportions; independent t-test to compare mean and Mann-Whitney U test to compare median. The prematurity adjusted composite BSID-III scores for cognitive, motor, language and social-emotional domains were calculated using the raw scores and scaled scores [25].

To examine the effect of ciKMC on the outcomes considered, univariable linear regression analysis was done as the initial step. This was followed by multivariable linear regression wherein potential confounding variables were included in the model. The choice of variables to be adjusted for was based on biological plausibility and/or on the statistical significance (*p* < 0.20) of their association with the outcome(s) of interest in the univariate analysis. Potential interaction between ciKMC and other variables, especially sex of the infant and wealth quintile, was examined by including interaction terms in the multivariable regression models.

Additionally, as an a priori decision, the continuous BSID-III and Infant Temperament Scale scores were categorized into quartiles to examine the effect of ciKMC using ordinal logistic regression. Odds ratios with 95% confidence intervals were used to report associations.

As an exploratory analysis, we also conducted a post-hoc equivalence testing of means of cognitive, language and motor scores across the two study groups. The purpose of this equivalence testing was to provide evidence to support the contention that ciKMC does not do harm in terms of neurodevelopment as there could be an argument that ciKMC improves survival at the cost of poorer neurodevelopment. We used Statgraphics Centurion Version 18.0 (http://www.statgraphics.com/centurion-xviii) statistical analysis software to perform the post-hoc equivalence testing using two one-sided tests of equivalence (TOST). The lower (Δ_L_) and upper (Δ_U)_ equivalence limits were set as − 3.0 and + 3.0 points respectively (1 SD =15 points for BSID; 3.0 points equate to 0.20 SD). Equivalence limits were set based on the discussion among the study investigators and with clinical psychologists. A difference of more than 3 points in the composite scores was considered to be clinically relevant. We defined “equivalence” as: Δ_L_ ≤ μ_1_-μ_2_ ≤ Δ_U_ where μ1 - μ2 represent the difference in mean scores among the two study groups. Null hypothesis considered was H0: μ_1_ - μ_2_ < Δ_L_ or μ_1_ - μ_2_ > Δ_U._

At a *p*-value of < 0.05, null hypothesis is rejected and “equivalence” is considered to be present.

## Results

**Fig. 1:**
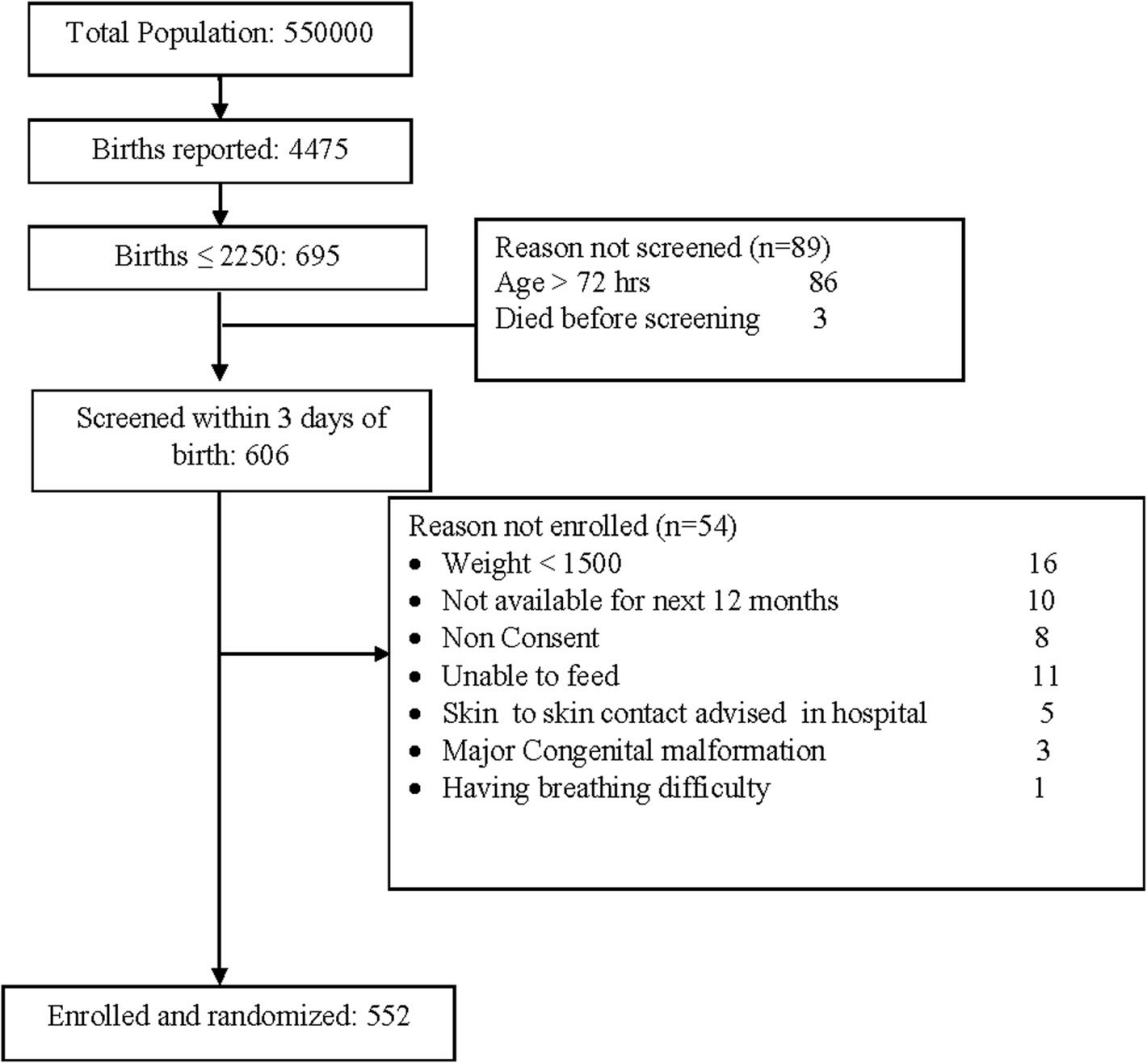
Trial profile

Figure 1 shows the trial profile. Among the 4475 reported births, 695 (15.5%) weighed ≤2250. Of these, 606 were screened within 72 h of birth and of them, 552 infants with birth weight ≥ 1500 g and ≤ 2250 g were enrolled in the study and randomized either into the intervention or control group (Fig. 1). At 6 and 12 months of follow up, 521 (94.4%) and 516 (93.5%) children, respectively, were available for evaluation. The baseline characteristics of the enrolled participants are described in Table 1. The baseline characteristics among the intervention and control groups were similar. The mean (SD) birth weight was 2051 (164) g in the intervention group and 2066 (169) g in the control group. The mean (SD) gestational age of the infants in the intervention group was 35.6 (1.9) weeks and 35.7 (2.0) weeks in the control group.

**Table 1:** Baseline characteristics of the primary trial population (*N* = 552)

Variables	ciKMC group (***N*** = 276)	Control group (***N*** = 276)
**HOUSEHOLD CHARACTERISTICS**		
**Wealth Quintiles**		
1 (Least poor)	53 (19.2)	57 (20.6)
2	50 (18.1)	62 (22.5)
3	59 (21.4)	51 (18.5)
4	55 (19.9)	55 (19.9)
5 (Poorest)	59 (21.4)	51 (18.5)
**Religion**		
Hindu	227 (82.2)	223 (80.8)
Muslim	48 (17.4)	50 (18.1)
Others^a^	1 (0.4)	3 (1.1)
**Social class**^**b**^		
General	64 (23.2)	73 (26.5)
Other Backward Class (OBC)	88 (31.9)	93 (33.7)
Scheduled Caste/Tribe (SC/ST)	124 (44.9)	110 (39.8)
**Type of family**		
Nuclear	67 (24.3)	73 (26.5)
Joint	209 (75.7)	203 (73.5)
*Mean number of family members (SD)*	8.22 (3.5)	8.30 (4.1)
**MATERNAL AND PATERNAL CHARACTERISTICS**		
**Mother’s age (in years)**		
< 20	32 (11.6)	26 (9.4)
20–29	218 (79.0)	233 (84.4)
≥ 30	26 (9.4)	17 (6.2)
*Mean maternal age (years; SD)*	23.14 (3.9)	22.95 (3.6)
**Mother’s education (years of schooling)**		
Illiterate (0)	110 (39.9)	96 (34.8)
Primary (1–5)	37 (13.4)	40 (14.5)
Secondary (6–12)	116 (42.0)	121 (43.8)
Higher than secondary (≥13)	13 (4.7)	19 (6.9)
*Median years of education (IQR)*	5 (0–9)	6.5 (0–10)
**Mother’s occupation**		
Employed outside home	6 (2.2)	4 (1.5)
Home maker	270 (97.8)	272 (98.5)
**Father’s age (in years)**		
< 20	5 (1.8)	4 (1.5)
20–29	204 (73.9)	212 (76.8)
≥ 30	67 (24.3)	60 (21.7)
*Mean father’s age (years; SD)*	26.70 (5.1)	26.16 (4.4)
**Father’s education (years of schooling)**		
Illiterate (0)	38 (13.8)	27 (9.7)
Primary (1–5)	50 (18.1)	43 (15.6)
Secondary (6–12)	155 (56.2)	163 (59.1)
Higher than secondary (≥13)	33 (11.9)	43 (15.6)
*Median years of education (IQR)*	8 (5–11)	9 (5–12)
**Father’s occupation**		
Employed in a government/private firm	106 (38.4)	119 (43.1)
Daily wage earner	73 (26.5)	53 (19.2)
Self-employed (own business/farming)	87 (31.5)	90 (32.6)
Unemployed	10 (3.6)	14 (5.1)
**BIRTH RELATED CHARACTERISTICS**		
**Place of delivery**		
Home	89 (32.2)	71 (25.7)
Government facility	134 (48.6)	149 (54.0)
Private facility	53 (19.2)	56 (20.3)
**Type of delivery**		
Normal	272 (98.5)	274 (99.3)
Caesarean section	4 (1.5)	2 (0.7)
**Birth order**		
1	107 (38.7)	99 (35.9)
2–3	115 (41.7)	129 (46.7)
≥ 4	54 (19.6)	48 (17.4)
*Median birth order (IQR)*	2 (1–3)	2 (1–3)
**Parity**		
Primiparous	107(38.8)	99 (35.9)
Multiparous	169 (61.2)	177 (64.1)
**INFANT CHARACTERISTICS**		
**Sex of the baby**		
Male	112 (40.6)	117 (42.4)
Female	164 (59.4)	159 (57.6)
**Mean birth weight** (grams; SD)	2051.3 (164.6)	2066.0 (169.4)
**Mean gestational age** (weeks; SD)	35.6 (1.9)	35.7 (2.0)

^a^Others: Christian/Sikh/Jain/Parsi/Zoroastrian/Buddhist/neo Buddhist; ^b^General- group that do not qualify for any of the positive discrimination schemes by Government of India (GOI), OBC- term used by the Government of India to classify castes which are socially and educationally disadvantaged, SC/ST- official designations given to groups of historically disadvantaged indigenous people in India; No statistically significant differences in the baseline characteristics between intervention and control group

Post-enrollment a total of 273 (98.9%) mothers in the intervention group and 10 (3.6%) mothers in the control group reported practice of SSC during the neonatal period. Within the intervention group, the median time to initiate ciKMC after birth was 48 h (IQR 48 to 72). Among these mothers, the mean duration of SSC practice was 27.9 (3.9) days with 8.7 (3.5) hours per day.

At 6 months of infant-age, findings from univariable and multivariable linear regression were not suggestive of any significant effect of the ciKMC on composite cognitive scores (difference-in-means 0.98; 95% CI − 1.30 to 3.26), language scores (difference-in-means-0.20; 95% CI − 1.99 to 1.58), motor scores (difference-in-means 0.83; 95% CI − 1.91 to 3.57), socio-emotional score (difference-in-means 0.10; 95% CI − 0.46 to 0.66) and infant temperament scores (difference-in-means − 2.01; 95% CI − 5.07 to 1.06) (Table 2). Ordinal regression analysis also did not show any significant effect of ciKMC on the above-mentioned outcomes (Supplementary Table 1). The results were similar at 12 months of infant-age where no significant effect of ciKMC was found on cognitive, language, motor, socio-emotional and infant temperament scores using multivariable or ordinal regression analysis (Table 2 and Supplementary Table 1). No significant interaction was observed in any of the models.

**Table 2:** Effect of ciKMC on Bayley Scales of Infant Development and Infant Temperament Scores at 6 and 12 months of infant age using linear regression

	At 6 months (***n*** = 521)			At 12 months (***n*** = 516)		
Outcome	Mean (SD)	Crude analysis	Multivariable analysis	Mean (SD)	Crude analysis	Multivariable analysis
		*Unadjusted ß*^*1*^*(95% CI)*	*Adjusted ß*^*1*^(95% CI)*		*Unadjusted ß*^*1*^*(95% CI)*	*Adjusted ß*^*1*^(95% CI)*
*Composite cognitive score*						
Control	95.49 (13.8)	Ref	Ref	101.98 (11.6)	Ref	Ref
ciKMC	96.47 (12.7)	0.98 (−1.30 to 3.26)	1.08 (−1.21 to 3.37)	102.19 (12.1)	0.21 (−1.84 to 2.27)	0.41 (− 1.58 to 2.40)
*Composite Language score*						
Control	88.51 (10.8)	Ref	Ref	85.39 (8.9)	Ref	Ref
ciKMC	88.30 (9.9)	-0.20 (− 1.99 to 1.58)	−0.03 (− 1.82 to 1.77)	84.48 (9.1)	− 0.90 (−2.47 to 0.67)	− 0.61 (− 2.10 to 0.89)
*Scaled Receptive language scores*						
Control	8.66 (2.5)	Ref	Ref	7.38 (1.5)	Ref	Ref
ciKMC	8.62 (2.3)	− 0.04 (− 0.46 to 0.38)	0.003 (− 0.41 to 0.42)	7.22 (1.6)	−0.16 (− 0.42 to 0.09)	− 0.13 (− 0.39 to 0.13)
*Scaled Expressive language scores*						
Control	7.34 (1.9)	Ref	Ref	7.54 (2.1)	Ref	Ref
ciKMC	7.30 (1.7)	−0.04 (− 0.35 to 0.27)	− 0.02 (− 0.33 to 0.29)	7.39 (1.9)	−0.15 (− 0.50 to 0.20)	−0.08 (− 0.42 to 0.25)
*Composite motor score*						
Control	95.91 (15.6)	Ref	Ref	90.64 (10.7)	Ref	Ref
ciKMC	96.74 (16.2)	0.83 (−1.91 to 3.57)	0.97 (− 1.71 to 3.66)	89.79 (10.2)	−0.85 (−2.65 to 0.96)	−0.75 (− 2.52 to 1.02)
*Scaled fine motor scores*						
Control	9.05 (2.9)	Ref	Ref	8.45 (1.5)	Ref	Ref
ciKMC	9.07 (3.1)	0.03 (−0.48 to 0.54)	0.03 (−0.48 to 0.54)	8.37 (1.5)	−0.08 (− 0.34 to 0.18)	−0.09 (− 0.35 to 0.17)
*Scaled gross motor scores*						
Control	9.53 (2.8)	Ref	Ref	8.42 (2.6)	Ref	Ref
ciKMC	9.80 (2.9)	0.27 (−0.24 to 0.78)	0.31 (−0.18 to 0.81)	8.22 (2.4)	−0.19 (− 0.62 to 0.24)	−0.14 (− 0.57 to 0.29)
*Composite socio-emotional score*						
Control	56.19 (3.1)	Ref	Ref	55.54 (1.6)	Ref	Ref
ciKMC	56.29 (3.4)	0.10 (−0.46 to 0.66)	0.11 (−0.44 to 0.66)	55.48 (1.5)	−0.06 (− 0.32 to 0.20)	−0.06 (− 0.33 to 0.20)
*Infant Temperament Score*						
Control	89.25 (17.8)	Ref	Ref	102.29 (15.2)	Ref	Ref
ciKMC	87.24 (17.9)	−2.01 (−5.07 to 1.06)	−1.70 (−4.85 to 1.47)	101.57 (14.9)	−0.72 (−3.33 to 1.88)	−0.49 (−3.15 to 2.18)

*Adjusted for socio-demographic characteristics (wealth quintile, religion, caste and number of family members); maternal characteristics (maternal age, maternal education); paternal characteristics (father’s age, father’s education); birth related characteristics (birth order, parity); infant characteristics (sex, birth weight, gestational age) and hospitalization in the neonatal period

^1^Reflects the difference in mean scores between the intervention and control groups of the trial

**Fig. 2:**
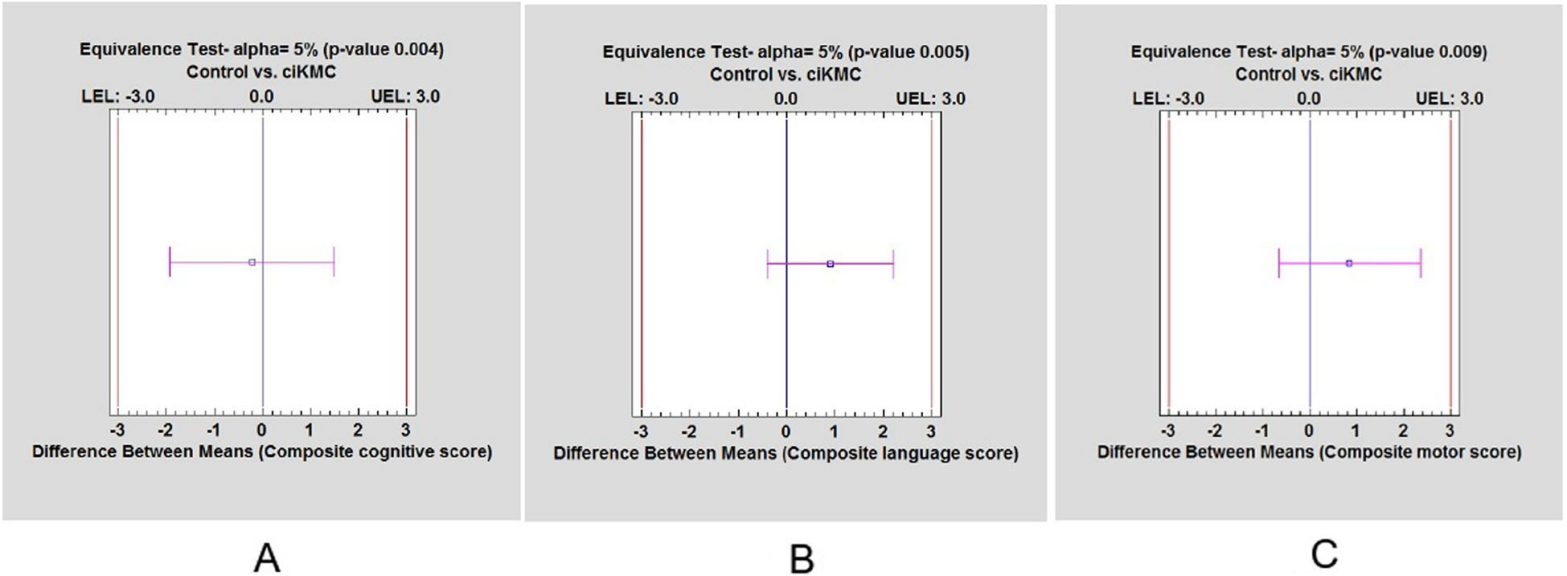
Plot showing equivalence of the (**a**) composite cognitive scores (**b**) language scores and (**c**) motor scores at 12 months of age among the two study groups

Results from regression analysis suggested no significant effect of the intervention on maternal PHQ 9 scores at 6 weeks and 6 months; maternal sense of competence at 6 weeks and 12 months; and PROCESS scores at 12 months of infant age (Table 3). Two one-sided tests of equivalence demonstrated that mean composite scores for cognitive, language and motor domains at 12 months among the two study groups were equivalent (Fig. 2).

**Table 3:** Effect of KMC on maternal PHQ-9 scores, mother-infant bonding, maternal sense of competence and home environment

Outcome	Mean (SD)	Univariate analysis	Multivariate analysis	***P***-value
		Unadjusted ß (95% CI)	Adjusted ß (95% CI)^a^	
**At 6 wks of infant age (*N*** **= 544)**				
Maternal PHQ-9 scores				
Control	1 (0–3)	Ref	Ref	
KMC	1 (0–3)	−0.08 (−0.56 to 0.39)	−0.12 (− 0.61 to 0.37)	0.642
Mother-infant bonding				
Control	86.44 (5.5)	Ref	Ref	
KMC	87.33 (5.2)	0.89 (−0.02 to 1.79)	0.82 (−0.08 to 1.72)	0.075
Maternal sense of competence				
Control	34.30 (3.6)	Ref	Ref	
KMC	34.75 (3.5)	0.45 (−0.15 to 1.04)	0.43 (−0.18 to 1.03)	0.169
**At 6 months of infant age (*N*** **= 544)**				
Maternal PHQ-9 scores				
Control	0 (0–0)	Ref	Ref	
KMC	0 (0–0)	−0.01 (−0.24 to 0.22)	−0.04 (− 0.26 to 0.19)	0.759
**At 12 months of infant age (*N*** **= 516)**				
Maternal sense of competence				
Control	37.13 (2.7)	Ref	Ref	
KMC	37.25 (2.7)	0.12 (−0.36 to 0.59)	0.14 (−0.34 to 0.62)	0.571
Home environment (PROCESS scores)				
Control	125.02 (16.5)	Ref	Ref	
KMC	123.01 (16.6)	−2.00 (−4.86 to 0.85)	−1.20 (−3.79 to 1.39)	0.363

β reflects the difference in mean scores between the intervention and control groups of the trial

^a^Adjusted for socio-demographic characteristics (wealth quintile, religion, caste and number of family members) ; maternal characteristics (maternal age, maternal education); paternal characteristics (father’s age, father’s education); birth related characteristics (birth order, parity); infant characteristics (sex, birth weight, gestational age) and hospitalization in the neonatal period

## Discussion

It is desirable that all LBW infants have access to a medical examination at birth and to a KMC program, with a high risk follow up to allow early and opportune intervention when any deviation is detected [21]. In settings where home-based deliveries are still occurring or access to a health facility is limited, community initiated KMC could improve survival. Even for settings with high rates of institutional birth, KMC initiation at health facilities may not happen and even if it does, it is possible that hospital to community continuum of care is not strengthened. In such situations, based on unpublished findings from the primary trial [21], community KMC programs could be cost effective if the health workers are trained in KMC and could promote it during their routine home follow up visits.

In a recently published randomized controlled trial by our organization, we documented that community initiated KMC (ciKMC) substantially improved survival in the neonatal period and in the first 6 months of life in LBW infants [21]. However, the impact of ciKMC on early child development, though plausible, is unclear. Our study aimed to generate evidence on the effects of KMC when initiated at the home (community initiated KMC) on child development. The study findings are not suggestive of any significant effect of ciKMC on child developmental outcomes in this specific group of stable LBW infants. Results of the post-hoc equivalence testing demonstrated that cognitive, language and motor scores among the study groups were equivalent thereby, providing evidence to indicate that ciKMC does not improve survival at the cost of poorer neurodevelopment. These findings are similar to a previous study that documented no statistically significant impact of KMC initiated at hospital on developmental outcomes at 12 months of corrected age in babies weighing ≤2000 g at birth [5].

Previous literature seem to suggest that the effect of KMC on child development may be greater in early preterm infants who are either less than 32–33 weeks of gestation or weight of < 1500 g [16]. The underlying rationale is that in these preterm infants, cerebral volume is not compromised [31, 32] and given the preserved brain anatomy and functionality, it might be possible that early interventions like KMC could accelerate neurodevelopment. This might be one of the reasons of not having a significant effect in our study as most (97%) of the LBW infants in our study were of gestational age ≥ 32 weeks with a mean of 36 weeks. In contrast to our findings, a previous controlled clinical trial by Bera et al. in LBW infants reported significant effects of hospital-initiated KMC on cognitive and motor development as assessed by Developmental Assessment Scale for Indian Infants (DASII) at 12 months of corrected-age [8]. In this study, the babies in the KMC arm had lower mean birth weight (1481.4 ± 363.6 g) and gestational age (33.3 ± 2.9 wks) compared to those in the control arm (birth weight:1848 ± 404.3 g; gestational age: 36.0 ± 2.6 wks) thereby increasing the probability of the observed positive effect of KMC. The difference in the results might be related to the use of different methods, including use of the culturally adapted DASII questionnaire in the Bera et al. study.

There can be a true possibility that ciKMC did not influence neurodevelopment in this very selected population of stable late preterm or term small for gestational age infants; however, the lack of significant effects of ciKMC in this study may also be due to the nature and timing of the measured outcomes. The mean (SD) composite cognitive, language and motor scores of the overall study infants i.e. from both intervention and control groups were 102.1 (11.8), 84.9 (9.1) and 90.2 (10.4) respectively. These obtained scores were similar to reported statistics from previously conducted studies in south Asian settings [33]. Given the low resource setting where this study was conducted, there may be possibility that any benefits of ciKMC on developmental outcomes is attenuated by the existing adversities. It is important to recognize that measurement of developmental outcomes during infancy is challenging. Although BSID-III scale has been used in young infants to document the effects of interventions on developmental outcomes and has been shown as a reliable tool for assessment, yet it may not be able to detect small differences in certain aspects of brain functioning by virtue of it being a global development assessment scale. For example, in a randomized trial of docosahexaenoic acid (DHA) supplementation in infancy, no differences between intervention groups were found on Bayley scores at age 18 months. However, differences were found in sustained attention using a visual habituation task at four, six, and nine months, indicating enhanced attention in infants who received higher doses of DHA [34]. Interestingly, a follow-up study of the same DHA trial found differences between intervention groups in several cognitive tasks at age five years [35]. This suggests that to evaluate effect of interventions, examination of individual cognitive systems is needed.

Prior studies have reported beneficial effects of KMC on autonomic and neuro-behavioral maturation and quality of sleep [11, 15, 16]. Moreover, through use of neuro-imaging and neuro-functional tests, KMC has also been shown to positively influence brain networks, synaptic efficacy [14] and increased volume of the left caudate nucleus which is believed to regulate fine motor skills [36]. It is possible that the effect of the intervention on outcomes such as cognition, language, motor development and infant temperament are subtle in infancy. The differences may manifest later in childhood that can be captured adequately using the age appropriate psychometric assessment and neuroimaging tools.

This argument gains impetus from the findings of a study where hospital-initiated KMC given during early infancy in preterm infants did not show any statistically significant differences in development scores at age of one year of age but was associated with reduced school absenteeism, hyperactivity, aggressiveness, externalization, and socio-deviant conduct of young adults after 20 years of enrollment [9, 12]. Considering the evidence that adults aged 20 years born small for gestational age at term had lower performances in subtests assessing attention and executive functions with lower volumes in the associated brain structures, it would be an important step forward to follow this cohort of infants and evaluate the effect of ciKMC in their long-term attentional performance [37]. We, therefore, plan to assess cognitive, higher executive functioning and early academic skills using validated tools with reliable psychometric properties in children from the primary cohort at ages 6–7 years. The primary study on 8402 babies had an overall comparatively earlier initiation time for KMC (30 h vs 48 h) and a higher daily dose (11.5 h vs 8.7 h) compared to our subsample of the first 552 infants. This is probably due to the improvement of intervention delivery in the study over time. In the follow-up study, our plan to select a subsample from the primary cohort that received the intervention (ciKMC) early and for a prolonged period. We conducted a post-hoc analysis to understand dose-response effect (≥8 h/day compared to < 8 h/day) of skin-to-skin-contact on neurodevelopmental outcomes and infant temperament scores. We did not find any significant effect; however, we were was not adequately powered for this analysis (Supplementary Table 2). We hope that the findings of the follow-up study would better inform us on the long-term benefits of ciKMC on child development.

The present study is probably the first attempt to assess the effect of ciKMC on child development. The study has several strengths including comprehensive and robust intervention delivery by an independent team that ensured optimal compliance; outcome assessment through an independent team of trained psychologists with quality checks. Further, attrition rates were very low and similar in the two trial groups. The limitations of this study include lack of reliable data on gestational age, and possibility of recall bias in reporting of the exact hours of skin-to-skin-contact per day between mother and baby. Moreover, because of restricting our study sample to infants weighing 1500- 2250 g at birth, we might have missed capturing any beneficial effects of ciKMC which is likely to be present in the very or extreme preterm infants.

## Conclusion

The present study found no statistically significant effect of ciKMC on cognitive, language, motor, socio-emotional development as assessed by BSID-III and temperament during infancy in this selected sample of stable late preterm or term small for gestational age infants. Long-term follow-up of the infants may provide critical insights on the effect of the intervention on cognitive capabilities. Despite not being able to show a significant effect of ciKMC on child development during infancy, it is still an important public health intervention for LBW infants in terms of improving neonatal and infant survival.

## Supplementary information

**Additional file 1: Table S1.** Effect of ciKMC on Bayley Scales of Infant Development and Infant Temperament Scores at 6 and 12 months of infant age using ordinal logistic regression. **Table S2.** Effect of the duration of SSC on BSID and Infant Temperament Scores at 12 months of infant age within the intervention group (*n* = 254).

## Data Availability

Data available on request to PI.

## Abbreviations

LBW: Low Birth Weight
KMC: Kangaroo Mother Care
WHO: World Health Organization
SSC: Skin-to-skin contact
ciKMC: Community-initiated Kangaroo Mother Care
RCT: Randomized controlled trial
CHRD SAS: Centre for Health Research and Development Society for Applied Studies
SNOSE: Serially numbered opaque sealed envelopes
ASHA: Accredited Social Health Activists
HBPNC: Home Based Post Natal Care
BSID-III: Bayley Scales of Infant and Toddler Development, 3rd Edition
ITS: Infant temperament scale
PHQ: Patient Health Questionnaire
PROCESS: Pediatric Review of Children’s Environmental Support and Stimulation
GCP: Good clinical practices
SD: Standard deviation
IQR: Inter-quartile range
USG: Ultrasound
OLR: Ordinal logistic regression
LMICs: Low middle income countries
DASII: Developmental Assessment Scale for Indian Infants
LAZ: Length for Age Z score

## References

[1] Katz J, Lee AC, Kozuki N, Lawn JE, Cousens S, Blencowe H, Ezzati M, Bhutta ZA, Marchant T, Willey BA et al: Mortality risk in preterm and small-for-gestational-age infants in low-income and middle-income countries: a pooled country analysis. Lancet 2013, 382(9890):417-425. doi: 10.1016/S0140-6736(13)60993-9. Epub 62013 Jun 60996. 10.1016/S0140-6736(13)60993-9 PMC3796350 23746775

[2] Christian P, Lee SE, Donahue Angel M, Adair LS, Arifeen SE, Ashorn P, Barros FC, Fall CH, Fawzi WW, Hao W *et al*: Risk of childhood undernutrition related to small-for-gestational age and preterm birth in low- and middle-income countries. Int J Epidemiol 2013, 42(5):1340-1355. doi: 10.1093/ije/dyt109. Epub 2013 Aug 1346. 10.1093/ije/dyt109 PMC3816349 23920141

[3] Allotey J, Zamora J, Cheong-See F, Kalidindi M, Arroyo-Manzano D, Asztalos E, van der Post J, Mol BW, Moore D, Birtles D (2018). Cognitive, motor, behavioural and academic performances of children born preterm: a meta-analysis and systematic review involving 64 061 children. Bjog.

[4] Whitaker AH, Feldman JF, Lorenz JM, Shen S, McNicholas F, Nieto M, McCulloch D, Pinto-Martin JA, Paneth N (2006). Motor and cognitive outcomes in nondisabled low-birth-weight adolescents: early determinants. Arch Pediatr Adolesc Med.

[5] Conde-Agudelo A, Diaz-Rossello JL. Kangaroo mother care to reduce morbidity and mortality in low birthweight infants. Cochrane Database Syst Rev. 2016;(8):CD002771. 10.1002/14651858.CD002771.pub4. 10.1002/14651858.CD002771.pub4 PMC6464509 27552521

[6] WHO (2003). Kangaroo mother care: a practical guide.

[7] Bear RJ, Mellor DJ (2017). Continuing education module-kangaroo mother care 2: potential beneficial impacts on brain development in premature infants. J Perinat Educ.

[8] Bera A, Ghosh J, Singh AK, Hazra A, Mukherjee S, Mukherjee R (2014). Effect of kangaroo mother care on growth and development of low birthweight babies up to 12 months of age: a controlled clinical trial. Acta Paediatr.

[9] Charpak N, Ruiz-Pelaez JG, Figueroa de CZ, Charpak Y (2001). A randomized, controlled trial of kangaroo mother care: results of follow-up at 1 year of corrected age. Pediatrics.

[10] Ohgi S, Fukuda M, Moriuchi H, Kusumoto T, Akiyama T, Nugent JK, Brazelton TB, Arisawa K, Takahashi T, Saitoh H (2002). Comparison of kangaroo care and standard care: behavioral organization, development, and temperament in healthy, low-birth-weight infants through 1 year. J Perinatol.

[11] Feldman R, Eidelman AI (2003). Skin-to-skin contact (kangaroo care) accelerates autonomic and neurobehavioural maturation in preterm infants. Dev Med Child Neurol.

[12] Charpak N, Tessier R, Ruiz JG, Hernandez JT, Uriza F, Villegas J, Nadeau L, Mercier C, Maheu F, Marin J, et al. Twenty-year Follow-up of Kangaroo Mother Care Versus Traditional Care. Pediatrics. 2017;139(1). 10.1542/peds.2016-2063 Epub 2016 Dec 2012. 10.1542/peds.2016-2063 27965377

[13] Ropars S, Tessier R, Charpak N, Uriza LF (2018). The long-term effects of the kangaroo mother care intervention on cognitive functioning: results from a longitudinal study. Dev Neuropsychol.

[14] Schneider C, Charpak N, Ruiz-Pelaez JG, Tessier R (2012). Cerebral motor function in very premature-at-birth adolescents: a brain stimulation exploration of kangaroo mother care effects. Acta Paediatr.

[15] Scher MS, Ludington-Hoe S, Kaffashi F, Johnson MW, Holditch-Davis D, Loparo KA (2009). Neurophysiologic assessment of brain maturation after an 8-week trial of skin-to-skin contact on preterm infants. Clin Neurophysiol.

[16] Feldman R, Rosenthal Z, Eidelman AI (2014). Maternal-preterm skin-to-skin contact enhances child physiologic organization and cognitive control across the first 10 years of life. Biol Psychiatry.

[17] GOI (2014). Kangaroo mother care and optimal feeding of low birth weight: Operational guidelines.

[18] Neogi SB, Chauhan M, Sharma J, Negandhi P, Sethy G (2016). Rolling out of kangaroo mother care in secondary level facilities in Bihar-Some experiences. Indian J Public Health.

[19] Bergh AM, de Graft-Johnson J, Khadka N, Om'Iniabohs A, Udani R, Pratomo H, De Leon-Mendoza S (2016). The three waves in implementation of facility-based kangaroo mother care: a multi-country case study from Asia. BMC Int Health Hum Rights.

[20] Sloan NL, Ahmed S, Mitra SN, Choudhury N, Chowdhury M, Rob U, Winikoff B (2008). Community-based kangaroo mother care to prevent neonatal and infant mortality: a randomized, controlled cluster trial. Pediatrics.

[21] Mazumder S, Taneja S, Dube B, Bhatia K, Ghosh R, Shekhar M, Sinha B, Bahl R, Martines J, Bhan MK, et al. Effect of community-initiated kangaroo mother care on survival of infants with low birthweight: a randomised controlled trial. Lancet (London, England). 2019. 10.1016/S0140-6736(19)32223-8 31590989

[22] Mazumder S, Upadhyay RP, Hill Z, Taneja S, Dube B, Kaur J, Shekhar M, Ghosh R, Bisht S, Martines JC (2018). Kangaroo mother care: using formative research to design an acceptable community intervention. BMC Public Health.

[23] Mazumder S, Taneja S, Dalpath SK, Gupta R, Dube B, Sinha B, Bhatia K, Yoshida S, Bahl R, Norheim OF (2017). Impact of community-initiated Kangaroo Mother Care on survival of low birth weight infants: study protocol for a randomized controlled trial. Trials.

[24] MOHFW (2014). Home based newborn care operational guidelines.

[25] Bayley N (2006). Bayley Scales of Infant and Toddler Development: Administration Manual, 3rd edition.

[26] Murray-Kolb LE, Rasmussen ZA, Scharf RJ, Rasheed MA, Svensen E, Seidman JC, Tofail F, Koshy B, Shrestha R, Maphula A (2014). The MAL-ED cohort study: methods and lessons learned when assessing early child development and caregiving mediators in infants and young children in 8 low- and middle-income countries. Clin Infect Dis.

[27] Kroenke K, Spitzer RL, Williams JB (2001). The PHQ-9: validity of a brief depression severity measure. J Gen Intern Med.

[28] Teti DM, Gelfand DM (1991). Behavioral competence among mothers of infants in the first year: the mediational role of maternal self-efficacy. Child Dev.

[29] Condon J, Corkindale C (1998). The assessment of parent-to-infant attachment: development of a self-report questionnaire instrument. J Reprod Infant Psychol.

[30] Casey PH, Barrett K, Bradley RH, Spiker D (1993). Pediatric clinical assessment of mother-child interaction: concurrent and predictive validity. J Dev Behav Pediatr.

[31] Ment LR, Hirtz D, Huppi PS (2009). Imaging biomarkers of outcome in the developing preterm brain. Lancet Neurol.

[32] Boardman JP, Counsell SJ, Rueckert D, Hajnal JV, Bhatia KK, Srinivasan L, Kapellou O, Aljabar P, Dyet LE, Rutherford MA (2007). Early growth in brain volume is preserved in the majority of preterm infants. Ann Neurol.

[33] Upadhyay RP, Naik G, Choudhary TS, Chowdhury R, Taneja S, Bhandari N, Martines JC, Bahl R, Bhan MK (2019). Cognitive and motor outcomes in children born low birth weight: a systematic review and meta-analysis of studies from South Asia. BMC Pediatr.

[34] Colombo J, Carlson SE, Cheatham CL, Fitzgerald-Gustafson KM, Kepler A, Doty T (2011). Long-chain polyunsaturated fatty acid supplementation in infancy reduces heart rate and positively affects distribution of attention. Pediatr Res.

[35] Colombo J, Carlson SE, Cheatham CL, Shaddy DJ, Kerling EH, Thodosoff JM, Gustafson KM, Brez C (2013). Long-term effects of LCPUFA supplementation on childhood cognitive outcomes. Am J Clin Nutr.

[36] Grahn JA, Parkinson JA, Owen AM (2008). The cognitive functions of the caudate nucleus. Prog Neurobiol.

[37] Suffren S, Angulo D, Ding Y, Reyes P, Marin J, Hernandez JT, Charpak N, Lodygensky GA (2017). Long-term attention deficits combined with subcortical and cortical structural central nervous system alterations in young adults born small for gestational age. Early Hum Dev.

