# Supplementary material for: Community initiated kangaroo mother care and early child development in low birth weight infants in India-a randomized controlled trial
Source: BMC Pediatr. 2020 Apr 4;20:150. doi: 10.1186/s12887-020-02046-4 (PMC7126178; doi:10.1186/s12887-020-02046-4)
Supplement: Supplementary file 1 — Additional file 1: Table S1. Effect of ciKMC on Bayley Scales of Infant Development and Infant Temperament Scores at 6 and 12 months of infant age using ordinal logistic regression. Table S2. Effect of the duration of SSC on BSID and Infant Temperament Scores at 12 months of infant age within the intervention group (n = 254). [file 12887_2020_2046_MOESM1_ESM.doc]

**Supplementary Table 1. Effect of ciKMC on Bayley Scales of Infant Development and Infant Temperament Scores at 6 and 12 months of infant age using ordinal logistic regression**

*Adjusted for socio-demographic characteristics (wealth quintile, religion, caste and number of family members) ; maternal characteristics (maternal age, maternal education); paternal characteristics (father’s age, father’s education); birth related characteristics (birth order, parity); infant characteristics (sex, birth weight, gestational age) and hospitalization in the neonatal period

| **Outcome** | **Univariate analysis** | **Multivariate analysis** | **P-value** |
| --- | --- | --- | --- |
|  | *Unadjusted OR (95% CI)* | *Adjusted OR (95% CI)** |  |
| **At 6 months of infant age (N=521)** | | | |
| *Composite cognitive score*  Control  ciKMC | Ref  1.13 (0.83 to 1.55) | Ref  1.10 (0.80 to 1.52) | 0.539 |
| *Composite Language score*  Control  ciKMC | Ref  0.92 (0.67 to 1.25) | Ref  0.93 (0.68 to 1.28) | 0.658 |
| *Composite motor score*  Control  ciKMC | Ref  1.13 (0.83 to 1.54) | Ref  1.14 (0.83 to 1.57) | 0.420 |
| *Composite socio-emotional score*  Control  ciKMC | Ref  0.94 (0.58 to 1.51) | Ref  0.89 (0.54 to 1.49) | 0.666 |
| *Infant Temperament Score*  Control  ciKMC | Ref  0.89 (0.66 to 1.22) | Ref  0.95 (0.69 to 1.30) | 0.736 |
| **At 12 months of infant age (N=516)** | | | |
| *Composite cognitive score*  Control  ciKMC | Ref  1.00 (0.73 to 1.37) | Ref  1.05 (0.76 to 1.46) | 0.747 |
| *Composite Language score*  Control  ciKMC | Ref  0.81 (0.59 to 1.10) | Ref  0.83 (0.60 to 1.14) | 0.255 |
| *Composite motor score*  Control  ciKMC | Ref  0.83 (0.61 to 1.14) | Ref  0.87 (0.63 to 1.20) | 0.410 |
| *Composite socio-emotional score*  Control  ciKMC | Ref  0.88 (0.50 to 1.56) | Ref  0.91 (0.50 to 1.66) | 0.768 |
| *Infant Temperament Score*  Control  ciKMC | Ref  0.94 (0.69 to 1.28) | Ref  0.96 (0.70 to 1.31) | 0.794 |

**Supplementary Table 2. Effect of the duration of SSC on BSID and Infant Temperament Scores at 12 months of infant age within the intervention group (n=254)**

*Adjusted for socio-demographic characteristics (wealth quintile, religion, caste and number of family members) ; maternal characteristics (maternal age, maternal education); paternal characteristics (father’s age, father’s education); birth related characteristics (birth order, parity); infant characteristics (sex, birth weight, gestational age) and hospitalization in the neonatal period

| **Duration of SSC in the intervention group** | *Composite Cognitive score* | *Composite Language score* | *Composite motor score* | *Composite socio-emotional score* | *Infant Temperament Score* |
| --- | --- | --- | --- | --- | --- |
|  | *Adjusted ß (95% CI)** | *Adjusted ß (95% CI)** | *Adjusted ß (95% CI)** | *Adjusted ß (95% CI)** | *Adjusted ß (95% CI)** |
| *Duration of SSC in days* | 0.25 (-0.17 to 0.66) | 0.20 (-0.11 to 0.51) | 0.01 (-0.35 to 0.36) | -0.01 (-0.07 to 0.04) | -0.30 (-0.84 to 0.25) |
| *Duration of SSC per day(hours)* | -0.24 (-0.68 to 0.20) | -0.18 (-0.51 to 0.15) | -0.36 (-0.73 to 0.02) | -0.02 (-0.07 to 0.04) | -0.20 (-0.77 to 0.38) |
| SSC < 8 hours/day  SSC ≥ 8 hours/day | Ref  -0.38 (-3.45 to 2.68) | Ref  0.24 (-2.04 to 2.53) | Ref  -0.25 (-2.86 to 2.37) | Ref  0.04 (-0.35 to 0.44) | Ref  -2.30 (-6.29 to 1.69) |
